# Steroid-Resistant Docetaxel-Induced Pneumonitis With Air Leak Syndrome: A Rare but Fatal Complication

**DOI:** 10.7759/cureus.101849

**Published:** 2026-01-19

**Authors:** Innes Turral, Rupak Kundu

**Affiliations:** 1 Critical Care, Yeovil Hospital, Yeovil, GBR

**Keywords:** air leak syndrome, cytotoxic lung injury, docetaxel-induced pneumonitis, oligometastatic prostate cancer, steroid-resistant pneumonitis

## Abstract

Docetaxel, a taxane commonly used in the treatment of metastatic prostate cancer, can occasionally cause pulmonary toxicity, most often presenting as interstitial pneumonitis. Although most patients respond favourably to corticosteroid therapy, a small subset develops a rapidly progressive, steroid-resistant pneumonitis characterised by diffuse alveolar damage, air leak syndromes, and high mortality. This report describes the case of a 74-year-old man with oligometastatic prostate adenocarcinoma who developed such a severe, steroid-refractory pneumonitis after his fifth cycle of docetaxel. Despite prompt drug withdrawal, escalation to high-dose intravenous methylprednisolone, and intensive supportive care, his respiratory status worsened. He subsequently developed pneumothorax, pneumomediastinum, and surgical emphysema. Extensive investigations ruled out alternative causes of respiratory deterioration such as infection, heart failure, vasculitis, and pulmonary embolism. This fatal case illustrates the rare but devastating cytotoxic form of docetaxel-induced lung injury and emphasises the importance of maintaining a high index of suspicion, initiating early diagnostic evaluation, and considering adjunctive immunomodulatory treatment strategies when corticosteroids fail.

## Introduction

Docetaxel is a semi-synthetic taxane derived from yew tree alkaloids that stabilises microtubules, inhibits mitosis, and remains a cornerstone treatment for metastatic prostate cancer [1]. Although generally well tolerated, pulmonary toxicity in the form of interstitial pneumonitis occurs in approximately 1-5% of patients [2]. In rare instances, severe alveolar damage may lead to air leak syndromes, such as pneumothorax or pneumomediastinum, reflecting alveolar rupture and extensive lung injury, and are associated with a significantly worse prognosis [3].

Docetaxel-induced pneumonitis (DIP) typically manifests between two and three weeks after administration, although cases have been reported as early as three days post-infusion [4]. The underlying mechanisms are thought to be multifactorial, involving immune-mediated hypersensitivity reactions, direct alveolar epithelial injury, and oxidative stress [5-7]. Most reported cases respond to corticosteroids and supportive care; however, a small proportion progress to severe, steroid-refractory disease associated with diffuse alveolar damage and high mortality [8]. There is scarce data supporting the use of immunomodulatory therapy beyond steroids in DIP, highlighting the need for further research into the mechanisms of lung injury and the development of an immunosuppressive strategy in such cases.

## Case presentation

A 74-year-old man with oligometastatic prostate adenocarcinoma (prostate-specific antigen 61.6 ng/mL, Gleason score 8) presented with worsening breathlessness, low-grade fever, general malaise, and muscle aches, symptoms that began 10 days after his fifth cycle of docetaxel chemotherapy (75 mg/m² IV). His medical history included coronary artery disease, hypothyroidism, depression, and an oesophagectomy for oesophageal cancer in 2023. He had quit smoking a decade earlier and was managing well with a Clinical Frailty Score of 3. He was receiving darolutamide alongside prednisolone (5 mg twice daily) as part of his cancer regimen, and his regular medications included levothyroxine and fluoxetine.

On admission, he was tachypnoeic at 28 breaths per minute, febrile at 38.1°C, and hypoxic despite 28% oxygen via nasal cannulae. Chest auscultation revealed fine crackles at both lung bases. Blood tests on admission showed elevated inflammatory markers (C-reactive protein (CRP) 79 mg/L, white cell count (WCC) 11 × 10⁹/L), though renal and liver function remained normal with a normal lactate (Table 1).

**Table 1:** Laboratory results at admission.

Test	Patient Value	Reference Range	Units
White Cell Count	11.43	4.0-10.0	×10⁹/L
Neutrophils	10.23	2.0-7.0	×10⁹/L
Lactate	1.1	0.5-2.0	mmol/L
C-reactive Protein	79	0-4	mg/L
Alkaline Phosphatase	107	30-130	U/L
Alanine Aminotransferase	15	0-50	U/L
Bilirubin	10	0-20	umol/L
Sodium	136	133-146	mmol/L
Potassium	4.2	3.5-5.3	mmol/L
Urea	4.6	2.5-7.8	mmol/L
Creatinine	97	59-104	umol/L
Procalcitonin	0.2	0.5- <2 (low risk for systemic infection)	µg/L

**Figure 1:**
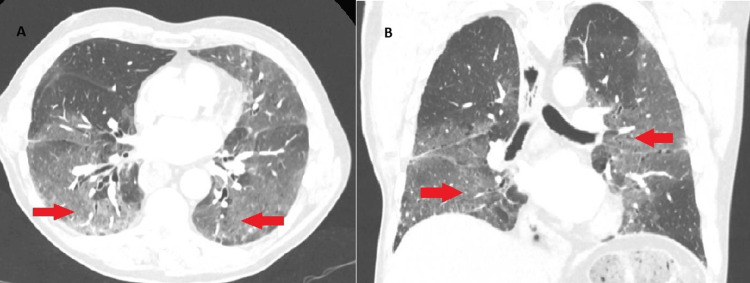
CT chest demonstrating diffuse bilateral ground glass opacities with interstitial thickening and sparing upper lobes highlighted with arrows. Axial (A) and coronal (B) views.

A chest X-ray revealed bilateral perihilar opacities, and he was admitted to the respiratory ward with a working diagnosis of community-acquired pneumonia. Empiric broad-spectrum antibiotics (ceftriaxone and doxycycline) were initiated. Four days later, a CT pulmonary angiogram ruled out pulmonary embolism but revealed widespread ground-glass opacities and interlobular septal thickening, predominantly in the mid and lower lung zones, findings consistent with interstitial pneumonitis (Figure 1).

**Figure 2:**
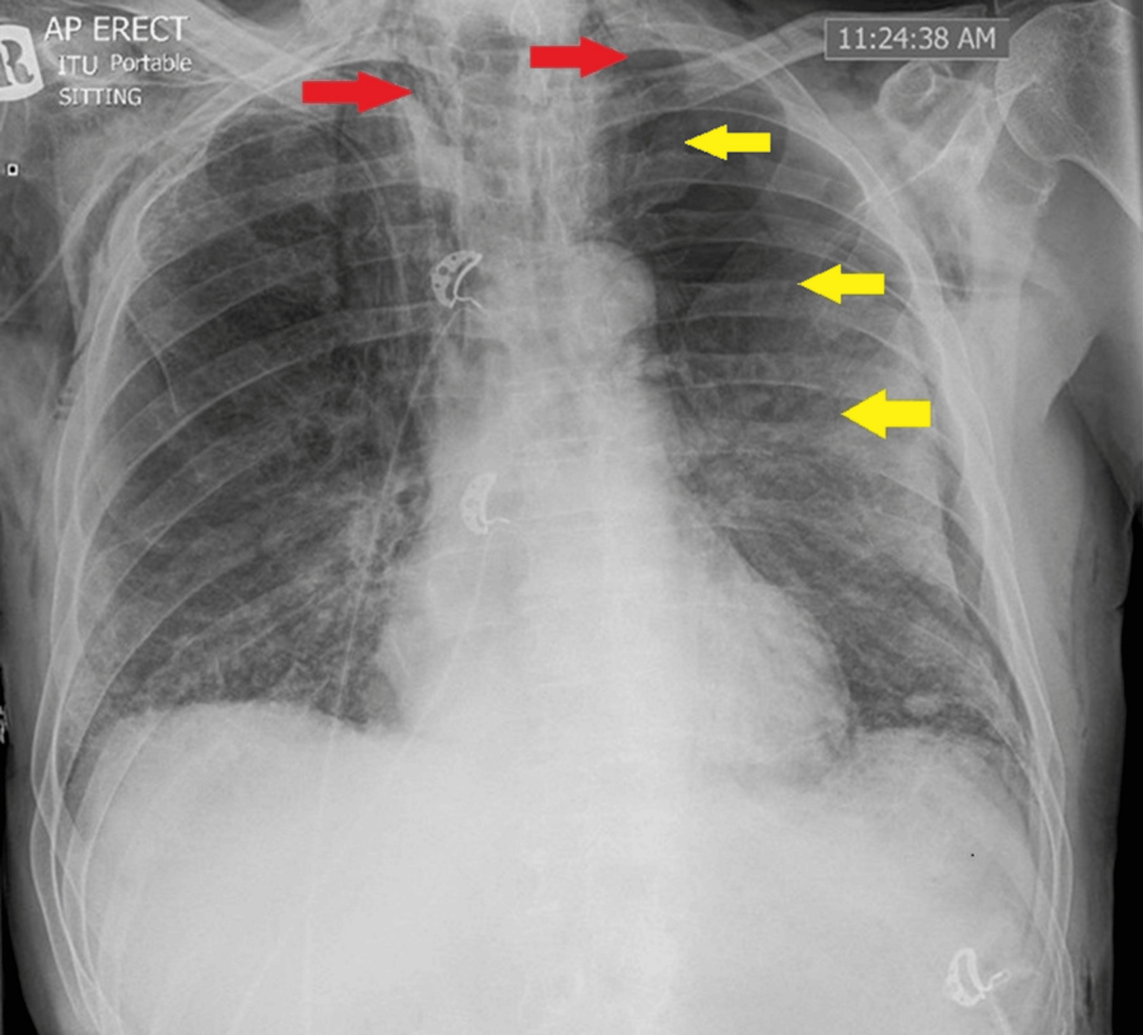
Portable chest X-ray showing subcutaneous emphysema (red arrows), pneumomediastinum, and left-sided pneumothorax (yellow arrows).

Seven days into admission, his respiratory failure worsened further, necessitating transfer to the intensive care unit (ICU) for high-flow nasal oxygen (HFNO) therapy. DIP was suspected, and he was started on IV hydrocortisone 50 mg four times daily for three days, followed by prednisolone 50 mg daily for six days. As hypoxia continued to worsen over the next couple of days, pulse methylprednisolone therapy (500 mg once daily) was commenced for three days. Comprehensive microbiological testing, including serial blood and sputum cultures, *Pneumocystis jirovecii* PCR, β-d-glucan, and *Aspergillus* antigen, was negative. Echocardiography showed preserved biventricular function with an ejection fraction of > 55% and no valvular disease. Autoimmune and vasculitis screens remained unremarkable. Inflammatory markers remained persistently high, with WCC ranging from 11-20 × 10⁹/L and CRP 79-295 mg/L. Serial procalcitonin levels remained low (0.20 µg/L), and Co-trimoxazole, which was started to cover possible pneumocystis pneumonia (PCP) and hospital-acquired pneumonia, was subsequently discontinued. He also developed air leak syndrome with surgical emphysema, a left-sided pneumothorax, and pneumomediastinum (Figure 2). The pneumothorax was unilateral, affecting the left lung, and a 12 Fr intercostal drain was placed on the left side.

Despite being hemodynamically stable, afebrile, and off vasopressors, his respiratory failure continued to worsen. He remained profoundly breathless despite maximal respiratory support with a fraction of inspired oxygen (FiO₂) of 100% on HFNO and up to 90% on non-invasive ventilation (NIV), alongside high-dose corticosteroids. A treatment escalation plan was established in consultation with the multidisciplinary team and family, determining that intubation and invasive mechanical ventilation would not be pursued due to their anticipated futility.

Three weeks into admission, following multidisciplinary discussions and in agreement with the family, further escalation was considered futile. Palliative measures were initiated, and the patient passed away peacefully the following day.

## Discussion

Docetaxel-associated lung injury is an uncommon but serious adverse effect, generally manifesting after two to four chemotherapy cycles, with clinical presentations ranging from mild cough to severe diffuse alveolar damage and acute respiratory distress syndrome (ARDS) [2,9]. Histopathology typically reveals lymphocytic alveolitis or interstitial fibrosis, consistent with an immune-mediated hypersensitivity reaction [10]. It is theorised to result from direct cytotoxic injury to alveolar epithelial and vascular endothelial cells rather than purely immune-mediated inflammation, leading to structural lung damage that progresses despite immunosuppressive therapy [8].

Our patient had some similar features with this pattern, exhibiting rapid symptom onset within two weeks from the last docetaxel infusion (albeit after the fifth dose), bilateral diffuse infiltrates on imaging, and progressive oxygen dependency despite escalated corticosteroid therapy. Although histological confirmation was not obtained, the development of pneumothorax, pneumomediastinum, and subcutaneous emphysema strongly suggests alveolar rupture secondary to diffuse alveolar damage, underscoring the cytotoxic nature of the injury. The most plausible mechanism for air leak syndrome in this case is extensive alveolar rupture secondary to diffuse alveolar damage. Cytotoxic injury from docetaxel likely weakened the alveolar-capillary barrier, and combined with high inspiratory pressures during respiratory distress and non-invasive ventilation, facilitated air dissection into the interstitium, mediastinum, and pleural space. It is plausible that baseline low-dose prednisolone therapy may have blunted early hypersensitivity inflammation, delayed classical presentation, and favoured cumulative epithelial cytotoxicity. 

Therapeutic strategies beyond corticosteroids remain empirical and largely anecdotal. Some case reports describe the use of cyclophosphamide or intravenous immunoglobulin (IVIG) to modulate persistent alveolar epithelial injury and fibrotic processes, extrapolating from other drug-induced interstitial lung diseases [11]. These therapies target molecular pathways involving transforming growth factor beta (TGF-β)-mediated fibroproliferation and oxidative stress cascades, which are implicated in sustained epithelial damage and steroid resistance. Nonetheless, robust clinical evidence supporting their efficacy in DIP is lacking, highlighting the need for further investigation.​

This case also illustrates the clinical challenge of distinguishing DIP from infectious pneumonias, as overlapping imaging and clinical features can delay initiation of high-dose corticosteroid therapy. High-resolution CT may have helped identify the aetiology earlier, enabling steroid administration, and whilst the patient could still tolerate lying flat with less oxygen support early in the patient's hospital journey. The use of IVIG and cyclosporine in addition to methylprednisolone was also considered in this case; however, the absence of evidence-based guidelines prevented the team from reaching consensus.

We propose the term 'docetaxel-induced acute lung injury (DIALI)' as a broader and more inclusive designation for the spectrum of acute inflammatory respiratory deterioration associated with docetaxel therapy. While 'docetaxel-induced pneumonitis (DIP)' is commonly used, it may not fully capture the diverse pathophysiological mechanisms involved, including direct alveolar toxicity, immune-mediated injury, and oxidative stress. We emphasise that this term is introduced as a conceptual suggestion for future consideration rather than formal classification, highlighting the varied clinical manifestations of taxane-induced pulmonary injury.

Clinical implications

Clinicians should maintain a high index of suspicion for DILAI in patients presenting with new or worsening respiratory symptoms during or after chemotherapy, even late in the treatment course. Early identification through high-resolution chest imaging and exclusion of infectious and cardiac causes are critical. Prompt discontinuation of docetaxel and initiation of corticosteroid therapy remain the cornerstone of management. However, in cases where no clinical improvement is observed within days of adequate steroid therapy, steroid-refractory cytotoxic alveolar injury should be considered. Emerging adjunctive treatments such as cyclophosphamide or intravenous immunoglobulin, targeting oxidative stress and fibroproliferation pathways, may offer benefit but lack robust evidence. Timely recognition and reporting of steroid-resistant cases are vital to improve understanding and develop more effective treatment strategies for this serious complication.

## Conclusions

This case illustrates a rare but devastating complication of docetaxel therapy, steroid-resistant interstitial pneumonitis, marked by rapid clinical decline, diffuse lung injury, and poor response to corticosteroids. The air leak syndrome supports a predominantly cytotoxic mechanism of alveolar damage rather than an immune-mediated process. Baseline low-dose corticosteroid may have contributed to the refractory disease course. While corticosteroids remain the cornerstone of management, evidence for alternative or adjuvant immunomodulators such as cyclophosphamide or IVIG is limited to isolated reports. These strategies should be regarded as investigational and considered only after thorough assessment of baseline lung condition, nutritional status, and overall clinical context. This case adds to the limited literature on steroid-refractory DILAI and highlights the urgent need for further research into its pathophysiology and effective targeted immunosuppressive therapies for this life-threatening condition.
